# Ultrasound and Elastosonographic Features of the Patellar Ligament in Dogs Affected by Cranial Cruciate Ligament Disease

**DOI:** 10.3390/vetsci11030126

**Published:** 2024-03-12

**Authors:** Luca Pennasilico, Antonella Volta, Sara Sassaroli, Caterina Di Bella, Valentina Riccio, Nicola Pilati, Adolfo Maria Tambella, Fabrizio Dini, Angela Palumbo Piccionello

**Affiliations:** 1 School of Bioscience and Veterinary Medicine, University of Camerino, 62024 Matelica, Italy; luca.pennasilico@unicam.it (L.P.); caterina.dibella@unicam.it (C.D.B.); valentina.riccio.dvm@gmail.com (V.R.); nicola.pilati@unicam.it (N.P.); adolfomaria.tambella@unicam.it (A.M.T.); fabrizio.dini@unicam.it (F.D.); angela.palumbo@unicam.it (A.P.P.); 2 Department of Veterinary Medicine Science, University of Parma, 43100 Parma, Italy; antonella.volta@unipr.it

**Keywords:** elastosonography, patellar ligament, stifle, cranial cruciate ligament, dog

## Abstract

**Simple Summary:**

In patients suffering from cranial cruciate ligament disease, the patellar ligament is loaded aphysiologically, resulting in increased stress. To date, however, there have been no studies describing the establishment of anatomical or functional damage to this ligament after cranial cruciate ligament disease. This study aims to evaluate the appearance of the patellar ligament using ultrasound and elastosonography in dogs affected by disease of the cranial cruciate ligament but not yet treated in order to understand whether signs of thickening and a reduction in elasticity increase over time between the day of ligament disease onset and the day of diagnosis and therefore persist before being treated with the surgical procedure of tibial plateau leveling osteotomy or tibial tuberosity advancement. The results suggest that as the time increases between the onset of cranial cruciate ligament disease and diagnosis and treatment, the patellar ligament progressively thickens and has a tendency to lose its elasticity.

**Abstract:**

This study aims to evaluate the morpho-functional change in the patellar ligament in dogs with cranial cruciate ligament disease. We hypothesized that it may show increased thickening and stiffness with increasing days from onset to diagnosis instead of trauma. Understanding this aspect has implications for the choice of timing for treating patients suffering from cranial cruciate ligament disease, as well as the contextualization of patellar ligament desmitis pictures after surgical treatment with tibial plateau leveling osteotomy or tibial tuberosity advancement. Thirty-three dogs affected by unilateral cranial cruciate disease were examined and divided into three groups based on the time elapsed from the onset of lameness to diagnosis: Group 1 (1–15 days), Group 2 (16–60 days), and Group 3 (over 60 days). Conventional B-mode ultrasonographic and elastosonographic examinations of the patellar ligament were performed without sedation for each dog. Upon ultrasonographic examination, all dogs showed modification in the echostructure of the patellar ligament. In addition, the patellar ligament tended to become harder with increasing days after disease, although there were no significant differences between groups. Our results show that as the time increases between the onset of cranial cruciate ligament disease and diagnosis and treatment, the patellar ligament progressively thickens and loses its elasticity.

## 1. Introduction

The cranial cruciate ligament (CCL) is an intraarticular structure in the stifle.

It originates in the caudomedial portion of the femur’s lateral condyle and the caudolateral portion of the femur’s intercondylar notch and is inserted diagonally in the cranial, medial and distal directions through the intercondylar notch to connect to the cranial intercondylar area of the tibia.

The CCL consists of two parts: the caudolateral and craniomedial bands. The first is larger, and it is taut in extension and becomes relaxed in flexion. The second is taut in both flexion and extension [1].

The CCL prevents the cranial translation and internal rotation of the tibia and the hyperextension of the stifle [2].

The synergic action of the CCL and caudal cruciate ligament avoids, to a lesser degree, the varus and valgus movements of the stifle joint. In addition, a human study revealed the presence of proprioceptive nervous endings within the ligament which could be involved in the proprioceptive reactions of the stifle joint to avoid excessive flexion and extension.

CCL disease is the most commonly acquired orthopedic disease and the first cause of hindlimb lameness in dogs [3,4]. The cause and pathogenesis of this disorder remain elusive. Abnormal anatomical conformation of the limb increased tibial plateau angle, overweight, reduction of elasticity and resistance to stress due to the patient’s age. Patient age, the presence of immune-mediated inflammatory diseases, breed predisposition, excessive acute limb loading, traumatic hyperextension and excessive internal rotation of the tibia are all pathogenetic hypotheses that have been postulated for overloading of the CCL [5,6].

Following the failure of the CCL, a cranial tibial thrust (CTT) is generated at every step of the subject affected by this pathology. This continuous cranial sliding of the tibia and the internal rotation of the tibia on the femur stress the other stifle’s anatomical structures, such as the meniscus and the medial collateral and patellar ligaments [7,8].

It is reported in the literature that 32% to 77% of dogs that have CCL disease also have meniscal injury [9,10,11]. Overweight, the chronicity of the injury to the CCL and complete, not just partial, disease of the ligament are factors that increase the incidence of meniscal damage [9,10,12]. The mechanism of the meniscal tear associated with CCL insufficiency relates to abnormal motion of the CCL–deficient joint [13,14]. The medial meniscus is usually affected by tears because there is a clear anatomical difference between the medial and lateral parts of the tibial plateau and the conformation of the medial and lateral menisci. In addition, the medial meniscus is firmly attached to the tibia and is compressed against the medial femoral condyle during cranial tibial thrust, while the lateral meniscus maintains a more neutral position [13].

Injury of the medial collateral ligament (MCL) in dogs is usually seen in conjunction with CCL disease [15,16]. The rotational stability of the stifle is guaranteed not only by the CCL but also by the medial and lateral collateral ligaments. In particular, internal rotation of the stifle is limited by the MCL and CCL itself. While rupture of the MCL alone is rare, following frequent disease of the CCL in dogs, an abnormal force acts on the MCL during flexion and the internal rotation of the tibia [17,18,19].

The patellar ligament is the tendon of the quadriceps femoris muscle that runs from the patella to the tibial tuberosity. As the final part of the tendon connects two bone structures, it is also referred to as the patellar ligament; however, its histological structure (86% collagen fiber type I) is more similar to a tendon than a ligament.

It is separated from the joint capsule by an infrapatellar fat pad. A small synovial bursa is often located between the ligament and the tibial tuberosity [20,21,22].

The patellar ligament is an important stabilizer of the stifle, and it is highly stressed in patients suffering from disease of the CCL [23,24].

To date, however, there have been no studies describing the establishment of anatomical or functional damage to this ligament after CCL deficiency.

The diagnosis of patellar ligament desmopathy is mainly clinical but should be investigated by means of instrumental investigations aimed at detecting the extent of the damage. Some different diagnostic imaging techniques have been described to evaluate alterations of the patellar ligament. X-ray examination allows one to see the general aspect of the ligament; however, it has limitations in subjects with a moderate or severe grade of inflammation [25]. Ultrasound and magnetic resonance imaging (MRI) are primarily used for the evaluation of the patellar ligament [26]. The first is cost-effective and widely used and has the advantage of being dynamic, unlike MRI. B-mode ultrasound has high sensitivity and specificity in detecting patellar tendinopathy, and it clearly shows ligament, paratenon, and periligamentous tissue. MRI, according to different sequences, is able to precisely identify the damage [27]. The advantages of MRI in patellar ligament desmopathy are related to its greater ability to pathologically image associated structures, such as the infrapatellar fat pad, that can coexist [28].

Warden et al. reported that ultrasonography was more accurate than MRI in confirming clinically diagnosed patellar tendinopathy. Ultrasound and MRI are both unable to provide indications of the mechanical and functional properties of the structures examined [28].

Elastosonography is an imaging technique complementary to ultrasound that evaluates the mechanical properties of tissue. The purpose of elastosonography is the qualitative or quantitative estimation of Young’s modulus, a physical parameter that identifies the stiffness of a material. This evaluation provides significant advantages:

- Distinguishes between different tissues based on their rigidity. This characteristic permits an immediate visual distinction using a graphical representation. Moreover, the contrast between the different tissues is generally high.
- Allows the detection of pathologies or disorders within the same tissue, especially in tissues whose function is closely related to their structure.
- Avoids the artifacts caused by the presence of ossification or mineralization (acoustic shadowing) [29].

In human medicine, elastosonography is used principally to diagnose thyroid, breast and prostate cancer and steatosis or liver fibrosis. Tissue stiffness is usually considered a biomarker of pathological lesions [30]. The biomechanical properties of tendons are associated with their function. For example, the Achilles tendon (the common calcaneal tendon) is substantially hard; in contrast, it is known that the patellar ligaments in clinically healthy dogs show highly elastic biomechanical properties [20]. The Achilles tendon acts as a stabilizer, while the patellar ligament allows the patella to glide in the trochlear groove in the proximal–distal direction [31]. In addition, there is a considerable difference between the range of motion of the stifle and crurotarsal joints. The first allows flexion–extension and rotation movement while the second only flexion-extension movement. 

Recently, a study evaluated the elasticity and softness of the patellar ligament in dogs using ultrasonographic and elastosonographic examinations. Overall, 89.3% of the 30 patellar ligaments examined were graded as soft or mostly soft, while the remaining 10.7% were classified as intermediate [32].

Two elastosonographic techniques are mainly described: strain and shear elastosonography. The first is also known as quasi-static or compression elastosonography, and it measures the deformation of tissue caused by external force and subsequently its ability to return to its original shape. This force is applied by an operator using a probe. The software generates a color map that expresses the relativity elasticity of different tissue structures. This map is represented in real time and is added to the ultrasound image of the tissues under examination.

The second is called dynamic elastosonography, and it evaluates the deformation of tissue caused by shear waves. The software analyzes the spread velocity of the ultrasound waves in the different tissues. A spread velocity bidimensional map is generated and superimposed onto a B-mode ultrasound image. The diffusion speed of the shear waves is correlated with the elasticity of the tissue, and it increases with the increasing rigidity of the tissues examined [33]. 

They are both described for the assessment of the patellar ligament [26].

Several studies have also proven that, following tibial plateau leveling osteotomy (TPLO) or tibial tuberosity advancement (TTA), between 50% and 61% of cases showed clinical and radiographic signs related to the thickening and tendinopathy of the patellar ligament. It is not yet clear whether the ligament, even before the tibia is treated with TPLO or TTA, is damaged with respect to the physiological picture [34,35].

The purpose of this study is to assess the appearance of the patellar ligament using ultrasound and elastosonography in dogs affected by disease of the CCL but not yet treated in order to understand whether the signs of thickening and a reduction in elasticity increase over time between the day of ligament disease onset and the day of diagnosis and therefore persist even before being treated with the surgical procedure of tibial plateau leveling osteotomy or tibial tuberosity advancement.

## 2. Materials and Methods

### 2.1. Ethics Statement

The Animal Welfare Body of the University of Camerino gave authorization for this clinical investigation to be carried out. The study was described in detail to all owners of the enrolled dogs, and they subsequently signed informed consent. 

### 2.2. Animals

Thirty-three dogs of various breeds with unilateral naturally occurring CCL disease were prospectively enrolled in the study. CCL disease was diagnosed based on craniocaudal instability of the stifle during orthopedic examination. All dogs underwent general, orthopedic and neurological physical examinations in order to exclude other concomitant diseases. In addition, their hematologic and biochemistry profiles were acquired. Patients affected by concomitant orthopedic, neurological or endocrine disease were excluded from the research, as well as dogs with contralateral CCL disease. A stifle joint that showed instability, clinical signs of disease (medial buttress, swelling, crepitus) and radiographic alterations were considered abnormal.

They were divided into three groups based on the time elapsed from the onset of lameness to diagnosis: Group 1 (1–15 days), Group 2 (16–60 days) and Group 3 (over 60 days).

The dogs were subjected to radiographic examination of the stifle and ultrasonographic and elastosonographic examinations of the patellar ligament.

### 2.3. Ultrasound and Elastosonographic Examination

The patients were positioned in lateral recumbency with the affected stifle up and in maximal manual passive flexion to deactivate the extensor mechanism. This position allowed us to avoid anisotropy of the fibrillar structures of the patellar ligament. With the dogs awake, a conventional B-mode ultrasonographic investigation of the patellar ligament was achieved using a MyLab Class C ultrasound machine (Esaote, Genova, Italy) equipped with a 12–18 MHz linear transducer (LA 435; Esaote, Genova, Italy). The cranial region was shaved from 2 cm distal to the tibial tuberosity to 1 cm proximal to the patella, and coupling gel was applied. A patellar ligament was reputed fit when the fibrillar echotexture was homogeneous, parallel and slightly broadened at the origin. Ligaments that showed ultrasonographic signs of pathologies, such as disrupted patterns, an increased cross-sectional diameter or internal mineralization, were considered abnormal.

The ligament thickness was calculated based on the longitudinal ultrasound images of the patellar ligament. The landmark for taking measurements was identified in the exact middle of the ligament measured from the most distal portion of its insertion into the patella to the most proximal insertion into the tibial tuberosity.

The elastosonographic images were obtained by applying light rhythmic pressure using the probe. The operator evaluated each patellar ligament twice and acquired only longitudinal sections. A color translucent map superimposed onto the B-mode images characterized the images.

Each color indicated the relative elasticity of the different structures compared with the mean elasticity of the entire area: blue (mostly hard), green (intermediate) and red (soft). Only images without artifacts were evaluated. The elastosonographic images were considered assessable when they showed:

- Consistency between the elastogram and the underlying B-mode ultrasound image;
- Blue coloration of the skin and dermal tissue because they are harder than the patellar ligament;
- Green coloration of the marker (green coil). Thus represents real-time feedback on the quality of the strain image acquisition.

The tissue elasticity was measured by calculating the percentage of softness and hardness using dedicated software (ElaXto, Esaote).

The ultrasonographic and elastosonographic evaluations were performed by a single expert radiologist.

The acquired data were compared between Groups 1, 2 and 3, and the group of dogs with CCL disease was compared to a group of dogs with healthy stifles that had already been studied [32].

After ultrasonographic and elastosonographic evaluations, all the dogs underwent X-ray examination with two projections in order to exclude concomitant stifle diseases and perform preoperative planning. Therefore, all patients were subjected to sedation to achieve a suitable degree of muscle relaxation and immobility during the diagnostic procedure. All dogs were premedicated with the same anesthetic protocol. Specifically, 2 μg/kg of dexmedetomidine and 0.2 mg/kg of butorphanol were administered intramuscularly. Subsequently, the cephalic vein was cannulated, and propofol (1–2 mg/kg) was administered as needed. Furthermore, additional oxygenation was ensured with the administration of pure oxygen via a face mask.

X-ray examination was performed using digital direct equipment (FDR D-EVO II, Fujifilm, Milano, Italy).

The tibial plateau angle (TPA) was measured in the mediolateral view with the stifle and tarsus joints flexed at 90°. The TPA was estimated at the insertion between the proximal tibial joint orientation line and the mechanical axis of the tibia in the sagittal plane. The angle between the proximal tibial joint orientation line and the axis perpendicular to the mechanical axis of the tibia was the TPA.

### 2.4. Statistical Analysis

The data were not normally distributed, so the Kruskal–Wallis nonparametric test was used to compare the patellar ligament thickness, hardness and softness values between groups. The hardness and softness values of the patellar ligament between the normal dogs and the dogs with CCL disease were compared using the Mann–Whitney U test. The correlation between the hardness and softness values and the time elapsed from the onset of lameness (days) was evaluated using Spearman’s test.

Statistical analysis was performed using the software IBM^®^ SPSS^®^ Amos v.26 (Bologna, Italy).

Statistical significance was set at a *p*-value < 0.05.

## 3. Results

### 3.1. Enrolled Patients

This research was conducted at the Veterinary Teaching Hospital of the University of Camerino (Matelica, Italy).

We examined 33 patellar ligaments from 33 dogs affected by unilateral cranial cruciate disease, 18 females and 15 males, belonging to different breeds of medium-sized and large dogs: five Labrador retrievers, four golden retrievers, four American Staffordshire terriers, three cani corsi italiani, three Siberian huskies, two rottweilers, one American pit bull terrier, one springer spaniel, one Braque Saint-Germain, one boxer, one German shepherd, one bracco italiano and six mixed-breed dogs. Eleven dogs belonged to Group 1, eight dogs to Group 2 and fourteen dogs to Group 3 (who had been lame for from 68 to 362 days). The mean weight was 32.1 ± 7.89 kg (Group 1 = 30.05 ± 6.5 kg; Group 2 = 33.85 ± 5.67 kg; Group 3 = 32.70 ± 9.90 kg; *p* > 0.05), and the mean age was 5.1 ± 2.61 years (Group 1 = 5.09 ± 2.87 years; Group 2 = 4.75 ± 3.15 years; Group 3 = 5.35 ± 2.20 years; *p* > 0.05). The tibial plateau angle showed no statical differences between groups (*p* > 0.05) (Group 1 = 26.16 ± 1.61°; Group 2 = 25.75 ± 1.48°; Group 3 = 26 ± 1.75°).

### 3.2. Ultrasonographic Examination

**Figure 1:**
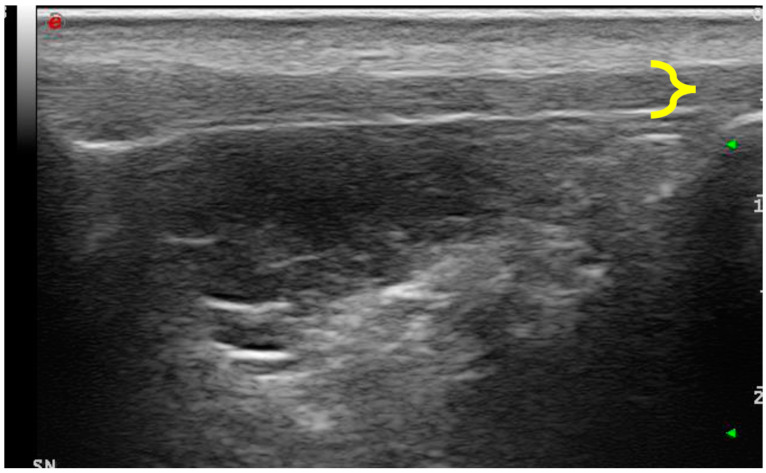
Ultrasonography of the patellar ligament of a dog affected by CCL disease. It appears to be thickened (delimited with yellow bracket) and characterized by an inhomogeneous echostructure.

**Figure 2:**
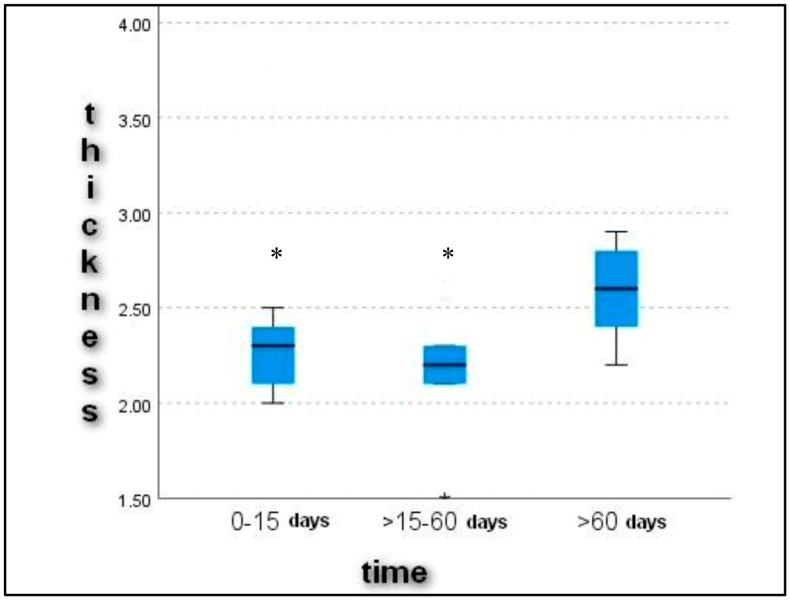
Graphical representation of patellar ligament thickness (millimeters) at different times between the onset of CCL disease and diagnosis and treatment. * shows a significant statistical difference from Group C (>60 days).

Upon ultrasonographic examination, all dogs showed alterations in the echostructure of the patellar ligament. The cross-sectional diameter of the patellar ligament was 2.4 ± 0.55 mm in Group 1, 2.1 ± 0.35 mm in Group 2 and 2.6 ± 0.41 mm in Group 3. The evaluation of the patellar ligament’s diameter revealed a progressive thickening of it with increasing time between the onset of lameness and diagnosis (Figure 1). These data expressed statistically significant differences (*p* < 0.05) in the comparison between Groups 2 (16–60 days) and 3 (>60 days) (*p* = 0.007) and between Groups 1 (0–15) and 3 (>60 days) (*p* = 0.037) (Figure 2).

### 3.3. Elastosonographic Examination

**Figure 3:**
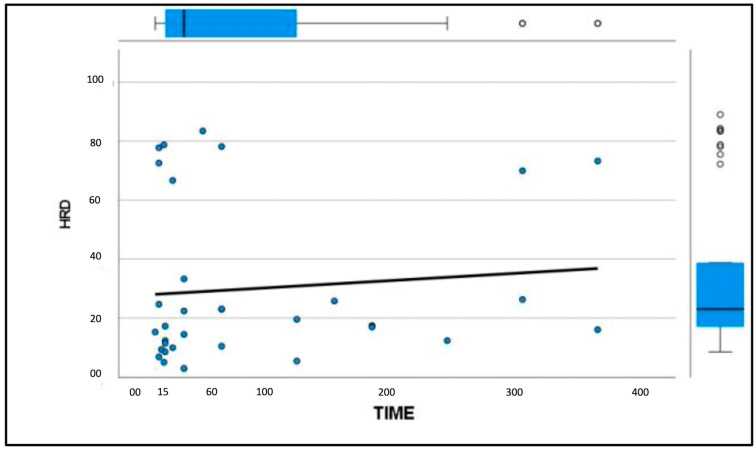
Graphical representation of patellar ligament hardness (%) with increasing time (days) between the onset of CCL disease and diagnosis.

**Figure 4:**
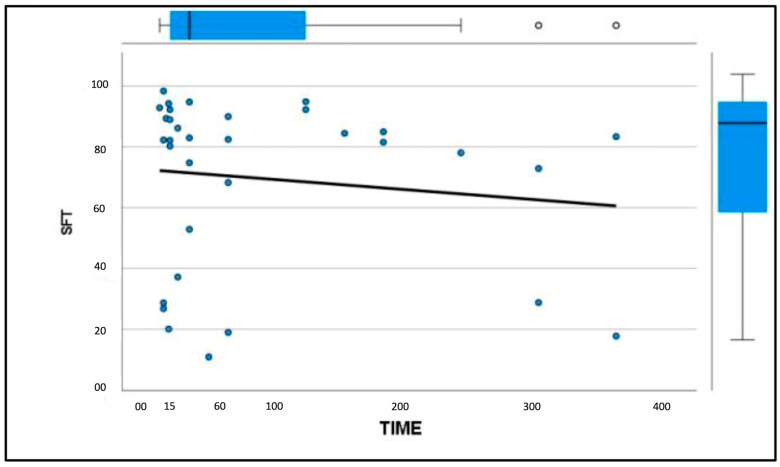
Graphical representation of patellar ligament softness (%) with increasing time (days) between the onset of CCL disease and diagnosis.

**Figure 5:**
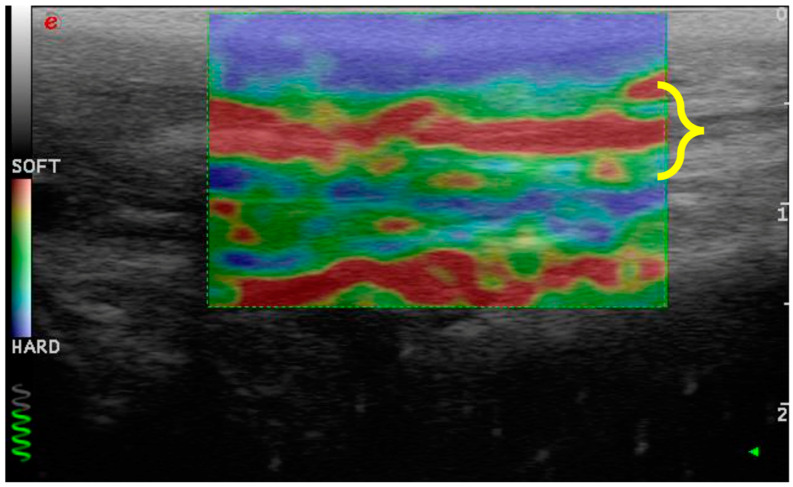
Elastosonography of the patellar ligament (delimited with the yellow bracket) of dogs withCCL disease. It shows harder areas (green) and soft areas (red).

**Figure 6:**
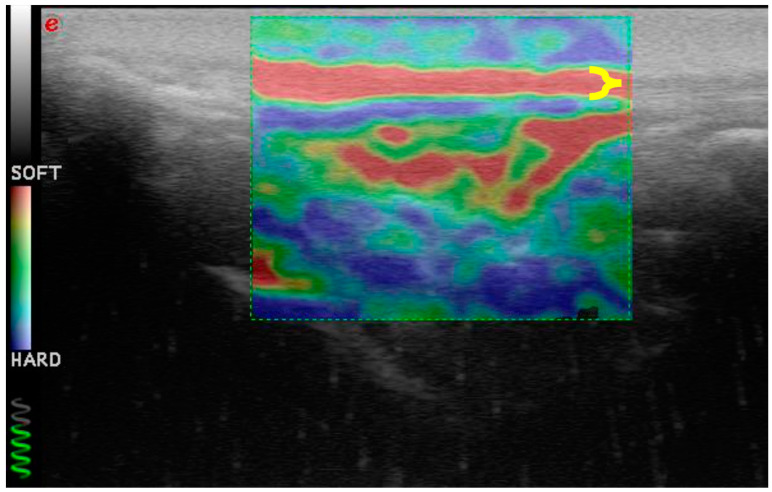
Elastosonography of the patellar ligament (delimited with yellow bracket) of a healthy dog. It appears homogeneously soft (red).

Similarly, the patellar ligament tended to become harder with increasing days after disease, although there were no significant differences between groups (Figure 3 and Figure 4). After comparison with the study group of healthy dogs, it was found that the patellar ligament of the dogs with CCL disease is harder than that of dogs with healthy stifles [36]. Specifically, the degree of hardness (HRD) was 25.5% in the healthy dogs and 30.1% in the CCL-disease-affected dogs, while the softness (SFT) was 74.5% in the healthy dogs and 69.9% in the affected group of dogs (Figure 5 and Figure 6).

## 4. Discussion

This is the first study to evaluate the alteration of the patellar ligament in subjects affected by CCL disease. The results showed an increased patellar ligament thickness and rigidity in dogs with chronic CCL disease. Specifically, there were significant differences between Groups 1 and 3 and between Groups 2 and 3 in the thickness of the patellar ligament. This means that alteration occurs gradually and is not related to acute trauma. 

Desmitis of the patellar ligament can be considered an overstress condition. The pathogenesis and precise identification of the intrinsic risk factors, in both humans and dogs, are not yet fully understood. However, some predisposing factors are reported in humans: obesity, age, dysmetria in the pelvic limbs, physical inactivity, malalignment of the pelvic limbs (such as genu valgus), sport activities which involve aggressive direction changes or jumping, hypotonia of the thigh muscles, repeated infiltration of corticosteroids and systemic diseases (such as diabetes or kidney failure) [37].

Patellar ligament disease is a rare disorder in dogs and horses, while it may have an incidence of 53% in human athletes [35]. This condition is commonly associated with thickening of the ligament without symptoms. In some cases, however, desmitis of the patellar ligament may appear with pain and lameness. Human and veterinary histological studies have reported the inconstant and variable presence of inflammatory cells in subjects affected by patellar ligament thickening. For this reason, the term desmopathy or tendinopathy is more appropriate because the patellar ligament undergoes a degenerative process [25,26].

Elastosonography is a feasible imaging modality for evaluating tissue strain and softness/stiffness in the tendons and ligaments. In this study, we used real-time strain elastosonography to study changes in the mechanical properties of the patellar ligament through the strain distribution resulting from tissue compression, even though it was reported that shear elastosonography is less operator-dependent than strain elastosonography in the evaluation of the patellar ligament [38]. Elastosonography is a quick and safe procedure, taking little time, often without the use of sedation, a contrast medium or both [36,39,40]. According to previous studies, we performed elastosonography in a few minutes. In our prior study, we showed that real-time elastosonography of the patellar ligament in healthy dogs was repeatable and reproducible. The ultrasonographic and elastosonographic evaluations were performed by a single expert radiologist in this study.

McCagherty et al. described the elastosonographic findings of the patellar ligament in healthy dogs in four different positions. The authors concluded that the most appropriate stifle angle for performing elastosonography was a natural standing angle [40]. We used maximal passive flexion, as reported in our previous study and in humans. The elastosonographic features of the patellar ligament in dogs affected by CCL disease were compared with those of healthy dogs, using the same stifle position to avoid inaccurate results [32].

Similar to the patellar ligament in humans, the normal canine patellar ligament shows a very soft elastogram [41,42]. Our study found, in dogs affected by CCL disease, significant progressive thickening of the patellar ligament and an increase in its hardness and a decrease in its softness with increasing time between the onset of lameness and diagnosis. Prior to our study, it was unclear whether and how the patellar ligament changes after CCL disease.

Veterinary studies have shown increased patellar ligament thickening in a large percentage of dogs undergoing tibial osteotomy (such as tibial plateau leveling osteotomy) following CCL disease [25,43]. Some of them have correlated this occurrence with the invasiveness of the surgical procedure itself, which could damage the ligament itself, or to the joint inspection methods [44].

Recently, only a few studies, mostly inspired by human medicine, have begun to hypothesize that TPLO changes the stifle biomechanics. Dan et al. described how TPLO reduces the stifle extensor mechanism’s moment arm, meaning that a greater force is required in the patellar ligament to achieve the same torque, so the patellar ligament is continuously under stress [34]. Zann et al. detected that the patellofemoral kinematics in TPLO-treated stifles were subtly different from normal ones, characterized by slight cranial shifting of the patella relative to the trochlear groove. These findings may provide further understanding of the extensor mechanism abnormalities associated with TPLO [44]. Guenego et al. saw that after TPLO, patellar height decrease and patellar ligament tendinosis occurred, regardless of the osteotomy position [45]. It had not yet been hypothesized that the disease of the cruciate ligament itself, causing continuous cranial tibial thrust in dogs (as opposed to humans), could cause constant stress on the patellar ligament and that therefore these biomechanical factors may be responsible for the thickening and increased hardness of the ligament in dogs even before undergoing surgery. An excessive angle of the tibial plateau could exacerbate the cranial tibial thrust that occurs after CCL disease and thereby stress the patellar ligament more. In this study, the TPA showed no statistical differences between groups, and it did not represent a variable in dogs with an increase in patellar ligament thickness and hardness.

The first limitation of this study is the small sample size, which was not calculated a priori using specific software, although the groups are homogeneous in terms of age, weight and the angle of the tibial plateau. Another limitation is related to the anatomical localization of the ultrasonographic evaluations. We measured the thickness and elasticity in the middle of the ligament; however, the main alterations probably occur in the distal portion at its attachment to the tibial tuberosity. In addition, the patellar ligament of the affected stifle joint was not compared with the contralateral equivalent in the same dog.

Understanding the results of our study regarding the thickness and hardness of the patellar ligament and the severity of stifle osteoarthrosis in correlation with the chronicity of the lesion before surgery can be of great use in formulating a prognosis in subjects who must undergo TPLO. 

Understanding this aspect could have implications for the choice of timing for treating patients suffering from disease of the CCL but also for the contextualization of the radiographic and ultrasound conditions of patellar ligament desmitis that are frequently observed in subjects undergoing TPLO and TTA.

Furthermore, these data will also act as a driving force to stimulate owners and veterinary colleagues to treat animals affected by CCL disease early to avoid ligament modifications resulting from chronic aberrant biomechanical stimuli. Undiagnosed desmopathy of the patellar ligament becomes more challenging to manage and sometimes requires a surgical approach. In severe cases, it is possible to observe rupture of the ligament [46]. 

## 5. Conclusions

In conclusion, understanding that as time increases between the onset of CCL disease and diagnosis and treatment, the patellar ligament progressively thickens and shows a tendency to lose its elasticity may be helpful in timing treatment and providing a possible correlation between the onset of postoperative desmitis and the condition of the ligament before surgery.

## Data Availability

The clinical data used to support the findings of this study are in cluded within the article.

## References

[1] Evans H.E., De Lahunta A. (2012). Miller’s Anatomy of the Dog.

[2] de Rooster H., Comerford E., Muir P. (2018). Morphology and function of the cruciate ligaments. Advances in the Canine Cranial Cruciate Ligament.

[3] Witsberger T.H., Villamil J.A., Schultz L.G., Hahn A.W., Cook J.L. (2008). Prevalence of and risk factors for hip dysplasia and cranial cruciate ligament deficiency in dogs. J. Am. Vet. Med. Assoc..

[4] Sellon D.C., Marcellin-Little D.J. (2022). Risk factors for cranial cruciate ligament rupture in dogs participating in canine agility. BMC Vet. Res..

[5] Griffon D.J. (2010). A review of the pathogenesis of canine cranial cruciate ligament disease as a basis for future preventive strategies. Vet. Surg..

[6] Kyllar M., Cížek P. (2018). Cranial cruciate ligament structure in relation to the tibial plateau slope and intercondylar notch width in dogs. J. Vet. Sci..

[7] Comerford E.J., Smith K., Hayashi K. (2011). Update on the aetiopathogenesis of canine cranial cruciate ligament disease. Vet. Comp. Orthop. Traumatol..

[8] Spinella G., Arcamone G., Valentini S. (2021). Cranial cruciate ligament rupture in dogs: Review on biomechanics, etiopathogenetic factors and rehabilitation. Vet Sci..

[9] Bennett D., May C. (1991). Meniscal damage associated with cruciate disease in the dog. J. Small Anim. Pract..

[10] Ralphs S.C., Whitney W.O. (2002). Arthroscopic evaluation of menisci in dogs with cranial cruciate ligament injuries: 100 cases (1999–2000). J. Am. Vet. Med. Assoc..

[11] Fitzpatrick N., Solano M. (2010). Predictive variables for complications after tibial plateau leveling osteotomy with stifle inspection by arthrotomy in 1000 consecutive dogs. Vet. Surg..

[12] Hayes G.M., Langley-Hobbs S.J., Jeffery N.D. (2010). Risk factors for medial meniscal injury in association with cranial cruciate ligament rupture. J. Small Anim. Pract..

[13] Franklin S.P., Gilley R.S., Palmer R.H. (2010). Meniscal injury in dogs with cranial cruciate ligament rupture. Compend Contin. Educ. Vet..

[14] Krupkova O., Smolders L., Wuertz-Kozak K., Cook J., Pozzi A. (2018). The pathobiology of the meniscus: A comparison between the human and dog. Front. Vet. Sci..

[15] Coppola M., Das S., Matthews G., Cantatore M., Czopowicz M., Silva L., McCarthy J., Fernandez-Salesa N., Lafuente P., Allan R. (2023). Multiligament stifle injury, a multicenter retrospective study in 26 dogs. Vet. Med. Sci..

[16] Bruce W.J. (1998). Multiple ligamentous injuries of the canine stifle joint: A study of 12 cases. J. Small Anim. Pract..

[17] Laing E.J. (1993). Collateral ligament injury and stifle luxation. Vet. Clin. N. Am. Small Anim. Pract..

[18] Palierne S., Blondel M., Vié K., Autefage A. (2022). Morphometric assessment of the medial collateral ligament of the canine stifle joint. Res. Vet. Sci..

[19] Aron D. (1998). Traumatic dislocation of the stifle joint: Treatment of 12 dogs and one cat. J. Am. Anim. Hosp. Assoc..

[20] Haut R.C., Lancaster R.L., DeCamp C.E. (1992). Mechanical properties of the canine patellar tendon: Some correlations with age and the content of collagen. J. Biomech..

[21] Lin T.W., Cardenas L., Soslowsky L.J. (2004). Biomechanics of tendon injury and repair. J. Biomech..

[22] Ricciardi M., Lenoci D. (2017). Comparative diagnostic imaging of a partial patellar ligament tear in a dog. Open Vet. J..

[23] Slocum B., Slocum T.D. (1993). Tibial plateau leveling osteotomy for repair of cranial cruciate ligament rupture in the canine. Vet. Clin. N. Am. Small Anim..

[24] Dennler R., Kipfer N.M., Tepic S., Hassig M., Montavon P.M. (2006). Inclination of the patellar ligament in relation to flexion angle in stifle joints of dogs without degenerative joint disease. Am. J. Vet. Res..

[25] Carey K., Aiken S.W., Di Resta G.R., Herr L.G., Monette S. (2005). Radiographic and clinical changes of the patellar tendon after tibial plateau leveling osteotomy 94 cases (2000–2003). Vet. Comp. Orthop. Traumatol..

[26] Pownder S.L., Hayashi K., Lin B.Q., Meyers K.N., Caserto B.G., Breighner R.E., Potter H.G., Koff M.F. (2021). Differences in the magnetic resonance imaging parameter T2* may be identified during the course of canine patellar tendon healing: A pilot study. Quant Imaging Med. Surg..

[27] Malmgaard-Clausen N.M., Tran P., Svensson R.B., Hansen P., Nybing J.D., Magnusson S.P., Kjaer M. (2021). Magnetic Resonance T2 * Is Increased in Patients with Early-Stage Achilles and Patellar Tendinopathy. J. Magn. Reson. Imaging.

[28] Warden S.J., Kiss Z.S., Malara F.A., Ooi A.B., Cook J.L., Crossley K.M. (2007). Comparative accuracy of magnetic resonance imaging and ultrasonography in confirming clinically diagnosed patellar tendinopathy. Am. J. Sports Med..

[29] Klauser A.S., Miyamoto H., Tamegger M., Faschingbauer R., Moriggl B., Klima G., Feutcher G.M., Kustlunger M., Jaschke W.R. (2013). Achilles tendon assessed with sonoelastography: Histologic agreement. Radiology.

[30] Sigrist R.M., Liau J., El Kaffas A., Chammas M.C., Willmann J.K. (2017). Ultrasound elastography: Review of techniques and clinical applications. Theranostics.

[31] Winnicki K., Ochała-Kłos A., Rutowicz B., Pękala P.A., Tomaszewski K.A. (2020). Functional anatomy, histology and biomechanics of the human Achilles tendon—A comprehensive review. Ann. Anat..

[32] Palumbo Piccionello A., Serrani D., Busoni V., Salvaggio A., Bonazzi M., Bergamino C., Volta A. (2018). Sonoelastographic features of the patellar ligament in clinically normal dogs. Vet. Comp. Orthop. Traumatol..

[33] Carlsen J.F., Pedersen M.R., Ewertsen C., Săftoiu A., Lönn L., Rafaelsen S.R., Nielsen M.B. (2015). A comparative study of strain and shear-wave elastography in an elasticity phantom. Am. J. Roentgenol..

[34] DeSandre-Robinson D.M., Tano C.A., Fiore K.L., Prytherch B. (2017). Radiographic evaluation and comparison of the patellar ligament following tibial plateau leveling osteotomy and tibial tuberosity advancement in dogs: 106 cases (2009–2012). J. Am. Vet. Med. Ass..

[35] Warden S.J., Brukner P. (2003). Patellar tendinopathy. Clin. Sports Med..

[36] Domenichini R., Pialat J.B., Podda A., Aubry S. (2017). Ultrasound elastography in tendon pathology: State of the art. Skeletal. Radiol..

[37] Van der Worp H., Van Ark M., Roerink S., Pepping G.J., Van Den Akker-Scheek I., Zwerver J. (2011). Risk factors for patellar tendinopathy: A systematic review of the literature. Br. J. Sports Med..

[38] Serrani D., Volta A., Cingolani F., Pennasilico L., Di Bella C., Bonazzi M., Salvaggio A., Palumbo A.P. (2021). Serial Ultrasonographic and Real- Time Elastosonographic Assessment of the Ovine Common Calcaneal Tendon, after an Experimentally Induced Tendinopathy. Vet. Sci..

[39] Del Signore F., De Dominicis S., Mastromatteo G., Simeoni F., Scapolo P.A., Tamburro R., Vignoli M. (2020). Sonoelastography of Normal Canine Common Calcaneal Tendon: Preliminary Results. Vet. Comp. Orthop. Traum..

[40] McCagherty J., Longo M., Pennington C., Liuti T., Morrison L.R., Brown H., Clements D.N. (2020). Effect of Stifle Flexion Angle on the Repeatability of Real-Time Elastosonography of the Patellar Ligament in Medium-to Large-Breed Dogs. Vet. Comp. Orthop. Traumatol..

[41] Porta F., Damjanov N., Galluccio F., Iagnocco A., Matucci-Cerinic M. (2014). Ultrasound elastography is a reproducible and feasible tool for the evaluation of the patellar tendon in healthy subjects. Int. J. Rheu..

[42] Zhang C., Duan L., Liu Q., Zhang W. (2020). Application of shear wave elastography and B-mode ultrasound in patellar tendinopathy after extracorporeal shockwave therapy. J. Med. Ultrason..

[43] Mattern K.L., Berry C.R., Peck J.N., De Haan J.J. (2006). Radiographic and ultrasonographic evaluation of the patellar ligament following tibial plateau leveling osteotomy. Vet. Radiol. Ultrasound..

[44] Zann G.J., Kim S.E., Tinga S., Pozzi A., Banks S.A. (2020). The effect of tibial plateau leveling osteotomy on patellofemoralkinematics in dogs: An in vivo study. Vet. Surg..

[45] Guénégo L., Vezzoni A., Vezzoni L. (2021). Comparison of tibial anatomical-mechanical axis angles and patellar positions between tibial plateau levelling osteotomy (TPLO) and modified cranial closing wedge osteotomy (AMA-based CCWO) for the treatment of cranial cruciate ligament disease in larjge dogs with tibial plateau slopes greater than 30° and clinically normal Labradors retrievers. BMC Vet. Res..

[46] Sundararajan S.R., Srikanth K.P., Rajasekaran S. (2013). Neglected patellar tendon ruptures—A simple modified reconstruction using hamstrings tendon graft. Intern. Orthop..

